# Alkaline peroxide pretreatment of corn stover: effects of biomass, peroxide, and enzyme loading and composition on yields of glucose and xylose

**DOI:** 10.1186/1754-6834-4-16

**Published:** 2011-06-09

**Authors:** Goutami Banerjee, Suzana Car, John S Scott-Craig, David B Hodge, Jonathan D Walton

**Affiliations:** 1 Department of Energy, Great Lakes Bioenergy Research Center, Michigan State University, East Lansing, MI, USA; 2 Department of Chemical Engineering and Materials Science, Michigan State University, East Lansing, MI, USA; 3 Department of Energy, Plant Research Laboratory, Michigan State University, East Lansing, MI, USA

## Abstract

**Background:**

Pretreatment is a critical step in the conversion of lignocellulose to fermentable sugars. Although many pretreatment processes are currently under investigation, none of them are entirely satisfactory in regard to effectiveness, cost, or environmental impact. The use of hydrogen peroxide at pH 11.5 (alkaline hydrogen peroxide (AHP)) was shown by Gould and coworkers to be an effective pretreatment of grass stovers and other plant materials in the context of animal nutrition and ethanol production. Our earlier experiments indicated that AHP performed well when compared against two other alkaline pretreatments. Here, we explored several key parameters to test the potential of AHP for further improvement relevant to lignocellulosic ethanol production.

**Results:**

The effects of biomass loading, hydrogen peroxide loading, residence time, and pH control were tested in combination with subsequent digestion with a commercial enzyme preparation, optimized mixtures of four commercial enzymes, or optimized synthetic mixtures of pure enzymes. AHP pretreatment was performed at room temperature (23°C) and atmospheric pressure, and after AHP pretreatment the biomass was neutralized with HCl but not washed before enzyme digestion. Standard enzyme digestion conditions were 0.2% glucan loading, 15 mg protein/g glucan, and 48 h digestion at 50°C. Higher pretreatment biomass loadings (10% to 20%) gave higher monomeric glucose (Glc) and xylose (Xyl) yields than the 2% loading used in earlier studies. An H_2_O_2 _loading of 0.25 g/g biomass was almost as effective as 0.5 g/g, but 0.125 g/g was significantly less effective. Optimized mixtures of four commercial enzymes substantially increased post-AHP-pretreatment enzymatic hydrolysis yields at all H_2_O_2 _concentrations compared to any single commercial enzyme. At a pretreatment biomass loading of 10% and an H_2_O_2 _loading of 0.5 g/g biomass, an optimized commercial mixture at total protein loadings of 8 or 15 mg/g glucan gave monomeric Glc yields of 83% or 95%, respectively. Yields of Glc and Xyl after pretreatment at a low hydrogen peroxide loading (0.125 g H_2_O_2_/g biomass) could be improved by extending the pretreatment residence time to 48 h and readjusting the pH to 11.5 every 6 h during the pretreatment. A Glc yield of 77% was obtained using a pretreatment of 15% biomass loading, 0.125 g H_2_O_2_/g biomass, and 48 h with pH adjustment, followed by digestion with an optimized commercial enzyme mixture at an enzyme loading of 15 mg protein/g glucan.

**Conclusions:**

Alkaline peroxide is an effective pretreatment for corn stover. Particular advantages are the use of reagents with low environmental impact and avoidance of special reaction chambers. Reasonable yields of monomeric Glc can be obtained at an H_2_O_2 _concentration one-quarter of that used in previous AHP research. Additional improvements in the AHP process, such as peroxide stabilization, peroxide recycling, and improved pH control, could lead to further improvements in AHP pretreatment.

## Background

The biochemical route for conversion of lignocellulose to biofuels is based on the fermentation of the sugars contained in plant cell wall polysaccharides. Generating fermentable sugars from lignocellulose can be achieved by a pretreatment coupled to an enzymatic depolymerization. Pretreatment chemistries can involve chemical modification, depolymerization, and/or solvation, resulting in physical redistribution of lignin and hemicelluloses and potentially altering cellulose crystallinity [1-4]. Many pilot and commercialization pretreatment processes are utilizing acidic pretreatments such as hot water, dilute acid (H_2_SO_4 _or SO_2_), and steam explosion. These are typically performed at temperatures in excess of 160°C and result in lignin melting and redistribution, hemicellulose solubilization and depolymerization, release of acetate from hemicelluloses, and the formation of furans from the acid-catalyzed dehydration of sugars. Other pretreatment alternatives include concentrated acid processes [5-7] and ionic liquids [8], which are based on decrystallizing cellulose; pretreatments based on ammonia [9,10]; a range of organosolv processes [11]; and delignifying alkaline and oxidative pretreatments such as wet oxidation [12-14] and alkaline hydrogen peroxide [15].

An ideal pretreatment would be effective (enable high sugar yields in a short time at low enzyme loading), simple (avoidance of multiple biomass handling steps), inexpensive (low capital equipment, energy, and chemical input requirements), and compatible with high biomass loadings. An ideal pretreatment would also minimize water consumption and not release or generate inhibitors of downstream operations (that is, enzyme digestion and fermentation). Current pretreatment methods are less than ideal in one or more of these characteristics. For example, although organic solvent/phosphoric acid mixtures and ionic liquids are very effective at rendering biomass amenable to enzymatic hydrolysis, they require high concentrations of expensive reagents [6-8]. Ammonia fiber expansion (AFEX), ammonia recycled percolation (ARP), SO_2_, and dilute acid operate at high pressure and/or high temperature and thus require capital-intensive and corrosion-resistant reactors. Acid pretreatment generates fermentation inhibitors, and lime requires long (4 week) residence times [3,13].

Pretreatment using hydrogen peroxide at pH 11.5, herein referred to as alkaline hydrogen peroxide (AHP), has been shown by Gould and colleagues to be effective for subsequent ruminant and *in vitro *enzyme digestibility [15-18]. AHP has been applied to corn stover [19,20], barley straw [21], wheat straw [22,23], bamboo [24], rice straw [25], softwood [26], sugarcane bagasse [27], Miscanthus [28] and other herbaceous and woody plants [29]. Saha and Cotta [21,22] reported that AHP compared favorably against dilute acid or lime pretreatment of barley straw for subsequent enzymatic sugar release, and that post-pretreatment washing was not required. AHP has been successfully operated in a continuous flow operation at high biomass loading (approximately 40% solids) and low H_2_O_2 _loading [30]. Nevertheless, AHP is relatively unstudied compared to other thermochemical pretreatments and has generally not been included in comparative studies and reviews [1-4].

In earlier experiments, we found that AHP compared favorably against dilute base (0.25% NaOH) or AFEX as a pretreatment for corn stover, Miscanthus, and switchgrass [20]. AHP pretreatment of corn stover yielded 18% more monomeric glucose (Glc) than AFEX (69% of total available Glc vs 52%) when the pretreated material was enzymatically hydrolyzed with a 16-component synthetic mixture at an enzyme loading of 15 mg protein/g glucan. Monomeric xylose (Xyl) yields were also higher from corn stover pretreated by AHP compared to AFEX (55% vs 41% of available Xyl) [20].

The AHP conditions used earlier [20] were essentially those developed by Gould [15,29], namely, a biomass loading of 2%, an H_2_O_2 _loading of 0.5 g/g biomass, and 24 h residence time at 23°C. In order to determine if the AHP process relevant to biomass conversion could be improved, we examined the effects of changing several AHP parameters on Glc and Xyl yields. We tested AHP-stover digestibility by single commercial enzyme cocktails, optimized mixtures of commercial cocktails, and synthetic mixtures of pure enzymes.

## Methods

### Biological materials

Corn stover (*Zea mays *L. Pioneer hybrid 36H56) was ground to pass a 5 mm screen and stored at room temperature. This material is called 'GLBRC stover' [20,31,32]. Before use, it was further ground in a Wiley mill to pass a screen size of 0.5 mm. No portion of the original material was discarded during any step of grinding. The final material contains 34.4% (w/w) Glc and 22.4% (w/w) Xyl. AFEX-pretreated material was ground in a Wiley mill to pass a screen size of 0.25 mm after pretreatment. For AFEX-pretreated corn stover, whether the grinding is performed before or after the AFEX treatment does not affect subsequent enzymatic digestibility [33].

Particle size has a large effect on enzymatic digestibility. Specifically, AFEX-pretreated corn stover ground to < 0.1 mm was shown in a side-by-side comparison to give absolute monomeric Glc yields that are 20 to 30% higher than AFEX-stover ground to pass a 0.5 mm screen size [32]. The material used in the current paper was ground to pass a screen size of 0.25-0.5 mm [32].

Commercial enzymes and purified enzymes were from the same lots described earlier [20,31,32].

### Pretreatments

For AHP, a solution of H_2_O_2 _(diluted from a commercial 30% stock, J.T. Baker ACS Reagent Grade) was titrated to pH 11.5 (± 0.2) with 5 M NaOH and mixed with the biomass. When comparing different biomass loadings, a fixed amount of biomass (1 g) was added to a fixed amount of H_2_O_2 _plus NaOH and a variable amount of water to give the desired final biomass loading. The experimental details of quantities and loadings of biomass, H_2_O_2_, NaOH, and water are given in Additional file 1, Table S1. Pretreatment biomass loadings are given as nominal % w/v, for example, 10% = 1 g biomass plus 10 ml pretreatment solution. All pretreatments were performed at 23°C in 125 ml flasks with shaking at 90 rpm for 24 h or 48 h. After AHP pretreatment, the biomass suspensions were neutralized to approximately pH 7 with concentrated HCl, treated with catalase to destroy residual H_2_O_2_, heated at 90°C for 15 min to inactivate the catalase, and lyophilized to dryness [20].

At the lowest H_2_O_2 _loading used in our experiments (0.125 g/g biomass), the pH tended to drift downward during the pretreatment incubation. In 'pH adjustment' experiments, the pH was maintained at 11.5 by addition of 5 M NaOH every 6 h (see Results). The amounts of NaOH added in these experiments are shown in Additional file 1, Table S2.

For AFEX pretreatment, GLBRC stover (5 mm particle size) was treated at an ammonia to biomass ratio of 2:1, a moisture content of 200%, a temperature of 150°C, and a residence time of 30 min. These AFEX conditions were more severe than those used previously [20,31,32].

### Enzymatic hydrolysis

The enzyme optimization platform GENPLAT was used to dispense stover slurries and enzymes. GENPLAT incorporates automated dispensing of stover slurry and enzymes into 96-well plates, statistical experimental design, and enzyme-mediated colorimetric determination of monomeric Glc and Xyl [20,31,32]. The final biomass concentration was 0.2% glucan (as monomeric Glc) suspended in 50 mM sodium citrate, pH 4.6. The mass of the salt introduced by the NaOH used for pretreatment and the HCl used for pH neutralization was taken into account when calculating glucan concentrations for the enzyme digestions (Additional file 1, Table S3) [20].

Optimization experiments with four-component commercial enzyme cocktail mixtures used an augmented quadratic experimental design and a fixed enzyme protein loading of 15 mg/g glucan. The lower limit of each of the commercial enzymes was set to 0%. The 11-component mixture experiments with purified enzymes also used a total protein loading of 15 mg/g glucan and an augmented quadratic design, which entailed 78 individual reactions. The lower limit for the 'core' enzymes (cellobiohydrolase 1 (CBH1), CBH2, endo-β1,4-glucanase 1 (EG1), β-glucosidase (BG), β-xylosidase (BX), and endo-β1,4-xylanase 3 (EX3)) were set to 4%, whereas the lower limit of all of the other 'accessory' proteins was set to 0% [20]. Each enzyme mixture was replicated once, sampled twice, and monomeric Glc and Xyl measured twice, for a total n = 8. For enzyme dose response experiments, the stover and enzyme mixtures (of a single proportional composition) were dispensed using the GENPLAT liquid handling robot, as stated in the figure legends.

### Effect of freeze drying on subsequent enzymatic hydrolysis

After AHP pretreatment and neutralization, biomass samples were typically lyophilized. The main reason for this was a concern about the stability of wet biomass during storage prior to the enzyme hydrolysis step. However, air drying biomass has been shown to adversely affect subsequent enzymatic digestibility [34], and therefore the effect of lyophilization on digestibility was tested. Corn stover (10 g) was placed in each of two 500 ml glass bottles at a final biomass loading of 10% and subjected to AHP pretreatment for 24 h at an H_2_O_2 _loading of 0.5 g/g biomass. One sample was then lyophilized whereas the other sample was subjected directly to enzymatic hydrolysis. The enzymatic hydrolysis conditions for both samples were 0.2% glucan loading, 15 mg enzyme/g glucan, pH 4.6, 48 h, and 50°C. The enzyme mixture comprised 64% Accellerase 1000, 9% Multifect Xylanase, and 27% Multifect Pectinase, which proportions were derived from small-scale experiments with the same pretreatment conditions (see Results). Digestibilities of the two samples were within 3% of each other (95 ± 2.2% for lyophilized vs 92 ± 2.0% for non-lyophilized; n = 3, ± 1 SD). We conclude that drying the pretreated biomass by lyophilization does not reduce subsequent sensitivity to enzymatic hydrolysis and is therefore an acceptable procedure.

## Results

### Influence of biomass, H_2_O_2_, and enzyme loadings

Initial AHP conditions were 2% biomass loading, 0.5 g H_2_O_2_/g biomass, pH 11.5, 24 h residence time, and 21°C to 24°C (that is, room temperature) [15,16,20,29]. In our experiments, corn stover pretreated in this way yielded 68.6% of total available monomeric Glc after digestion for 48 h with Accellerase 1000 at an enzyme loading of 15 mg/g glucan [20].

To determine if AHP could be further improved, we initially tested the influence of biomass loading. In this and all other experiments, the biomass was not washed or otherwise fractionated after pretreatment. That is, the entire material was freeze dried after AHP treatment and used as the substrate for enzymatic hydrolysis. The calculations of glucan content take into consideration the masses of the added salt (as NaOH plus HCl).

**Figure 1:**
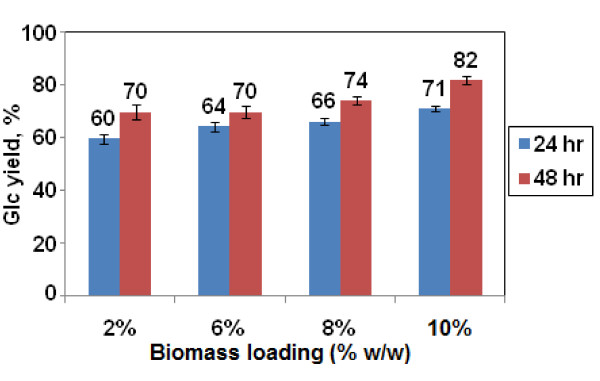
**Effect of pretreatment biomass loading on monomeric glucose (Glc) yield**. After pretreatment for 24 h at the indicated biomass loadings at 0.5 g H_2_O_2_/g biomass, the biomass was neutralized, diluted to 0.2% glucan, and digested with Accellerase 1000 (15 mg/g glucan) for 24 or 48 h. Error bars represent ± 1 SD (n = 8).

On increasing the biomass loading from 2% to 10% (see Additional file 1, Table S1 for the details of the reaction recipes), monomeric Glc release increased from 70% to 82% after 48 h digestion with Accellerase 1000 (Figure 1). That is, increasing the biomass loading increased the effectiveness of AHP. These experiments were performed by reducing the water content of the reaction mixtures while maintaining the H_2_O_2 _loading per g biomass constant, and therefore the molar concentrations of H_2_O_2 _and NaOH in the liquid phase increased with increasing biomass loading. The use of higher biomass loadings with these conditions is thus advantageous in terms of water consumption and digestibility but does not consume less H_2_O_2 _or NaOH per unit of treated biomass.

**Figure 2:**
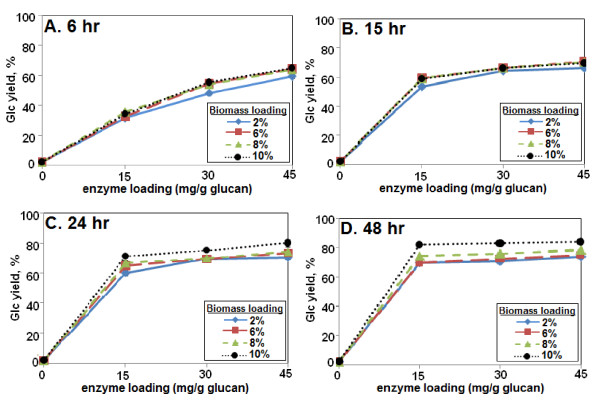
**Effect of pretreatment biomass loading, enzyme loading, and hydrolysis time on monomeric glucose (Glc) yields**. Biomass loadings ranged from 2% to 10%, enzyme loadings from 0 to 45 mg/g glucan, and digestion times from 6 to 48 h. Glc yield values indicate yield as a percentage of total measured monomeric Glc content of the biomass. The enzyme was Accellerase 1000.

Enzyme digestibility of biomass pretreated at different loadings was studied in more detail as a function of enzyme loading and digestion time. At 6 h, Glc yields were similar for all biomass loadings and were close to linear as a function of enzyme loading up to 45 mg/g glucan (Figure 2). By 48 h, Glc yields plateaued above an Accellerase 1000 loading of approximately 15 mg/g glucan (the lowest enzyme loading tested in this experiment), resulting in a Glc yield of approximately 82% (Figure 2).

**Figure 3:**
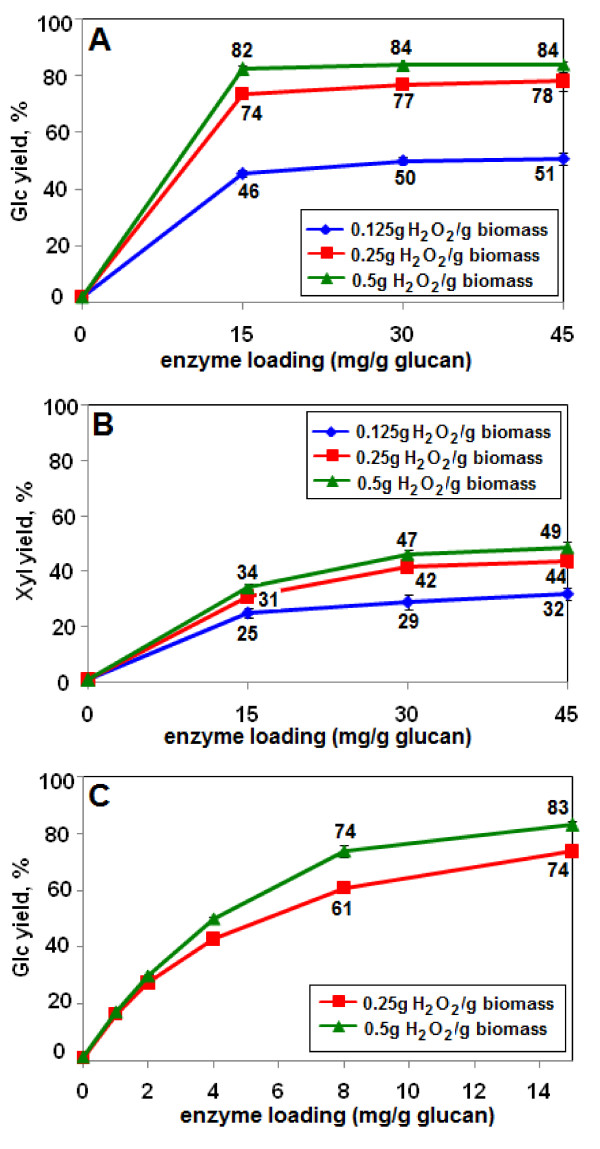
**Glucose (Glc) and xylose (Xyl) yields as a function of H_2_O_2 _and enzyme loading**. In panels **(a) **and **(b)**, the enzyme loadings ranged from 0 to 45 mg/g glucan. In panel **(c)**, the enzyme loading ranged from 0 to 15 mg/g glucan. Pretreatment biomass loading was 10%, residence time was 24 h, and enzyme digestion time was 48 h. Glc (a and c) and Xyl (b) yield values indicate yield as a percentage of total measured monomeric Glc and Xyl content of the biomass. Numbers next to the data points are the Glc or Xyl yields (y axis values). The enzyme was Accellerase 1000.

The effect of lower H_2_O_2 _loadings was then tested. An H_2_O_2 _loading of 0.25 g/g biomass was almost as effective for Glc release as 0.5 g/g (74% Glc vs 82%). However, 0.125 g/g was much less effective (that is, 46% Glc release) (Figure 3a). A similar trend of decreasing yield with decreasing H_2_O_2 _loading was seen for Xyl (Figure 3b).

The lowest enzyme loading tested in these experiments was 15 mg/g glucan, which for the higher H_2_O_2 _loadings at 48 h was already at or near a plateau of maximum Glc release (Figures 2d and 3a). Therefore, lower enzyme loadings were examined. Glc yields declined at enzyme loadings below approximately 15 mg/g glucan (from 83% Glc at 15 mg/g glucan to 74% at 8 mg/g) (Figure 3c). This indicates that the lowest enzyme loading that gave maximum Glc yield was, in fact, approximately 15 mg/g glucan (Figures 2d and 3a).

### Optimization of commercial enzyme cocktail mixtures for different H_2_O_2 _loadings

A critical feature of an effective pretreatment is high sugar yields at low enzyme loadings [4]. Accellerase 1000 is rich in cellulases but relatively deficient in xylanases [20,35]. To test the effectiveness of AHP in combination with a superior enzyme cocktail, a mixture of four commercial enzymes (Accellerase 1000, Multifect Xylanase, Multifect Pectinase, and Novozyme 188) was optimized using GENPLAT. The total enzyme loading was fixed at 15 mg/g glucan. Proportions of the four enzymes were independently optimized for each of three H_2_O_2 _concentrations (0.5, 0.25, and 0.125 g/g biomass). An optimized mixture of the four enzymes was superior to Accellerase 1000 alone at all H_2_O_2 _loadings. At 0.125 g H_2_O_2_/g biomass, monomeric Glc yields improved from 46% to 55% compared to Accellerase 1000 alone, and at 0.25 g/g biomass, Glc yields improved from 74% to 83% (Figure 3a, Table 1). At 0.5 g H_2_O_2_/g biomass, Glc yields increased from 82% to 95%. Monomeric Xyl yields, optimized only with 0.5 g H_2_O_2_/g, increased from 34% with Accellerase 1000 alone to 75% with an optimized mixture of the four enzymes (compare Figure 3b and Table 1).

**Table 1:** Optimization of mixtures of four commercial enzymes for monomeric glucose (Glc) and xylose (Xyl) release from corn stover pretreated by alkaline hydrogen peroxide (AHP) or ammonia fiber expansion (AFEX)

Pretreatment	Optimization	Optimal enzyme proportions (%)				Glc or Xyl yield (%)	
	
		Acc 1000	Multifect xylanase	Multifect pectinase	Novozyme 188	MP	Exptl
0.125 g H_2_O_2_/g biomass	Glc	59	25	16	0	54.0	55.0 ± 1.3

0.25 g H_2_O_2_/g biomass	Glc	63	14	21	2	86.0	83.0 ± 1.0

0.5 g H_2_O_2_/g biomass	Glc	64	9	27	0	99.6	95.0 ± 2.4
	
	Xyl	36	19	45	0	74.0	75.1 ± 0.9

AFEX	Glc	66	34	0	0	62.0	61.5 ± 1.5
	
	Xyl	40	10	50	0	55.7	53.4 ± 2.1

For all AHP experiments, pretreatment biomass loading was 10% and pretreatment time was 24 h with no pH adjustment during the pretreatment. For AFEX pretreatment conditions see Methods. Enzyme loading was 15 mg/g glucan and digestion time was 48 h. Glc and Xyl values indicate yield as a percentage of total measured monomeric Glc and Xyl content of the biomass. Raw data and statistical analysis are shown in Additional file 1, Supplementary Tables S4-S8.

Acc 1000 = Accellerase 1000; Exptl = experimental results (mean ± 1 SD, n = 8); MP = model prediction.

**Figure 4:**
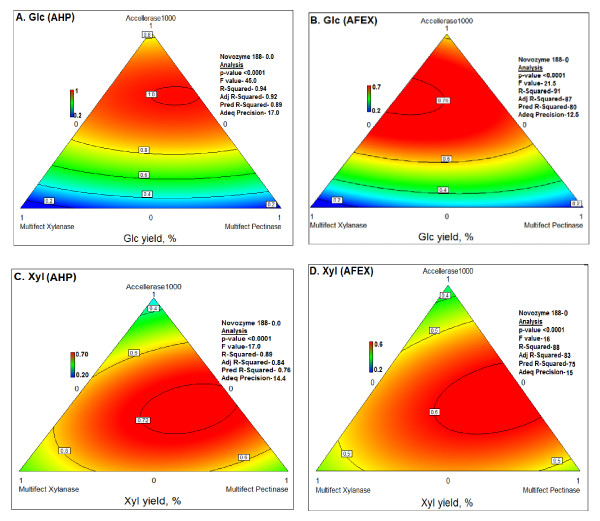
**Ternary diagrams for optimization of mixtures of four commercial enzymes for release of glucose (Glc; (a) and (b)) or xylose (Xyl; (c) and (d)) from alkaline hydrogen peroxide (AHP)-pretreated ((a) and (c)) or ammonia fiber expansion (AFEX)-pretreated ((b) and (d)) corn stover**. Glc and Xyl yield values indicate yield as a percentage of total measured monomeric Glc and Xyl content of the biomass. The data on which the ternary diagrams are based are from the same experiment shown in Table 1.

The corresponding ternary diagrams for optimized mixtures of the four commercial enzymes on biomass treated with 0.5 g H_2_O_2_/g are shown in Figure 4. Compared to the Glc ternary diagrams, the Xyl diagrams (Figure 4c,d) evidenced a relatively flat topology, that is, multiple solutions gave optimal Xyl release within a few percent of each other. This is reasonable considering that all four of the commercial enzymes contain xylanase activity, and therefore Xyl yield is not as strongly dependent as Glc yield on their relative proportions.

**Figure 5:**
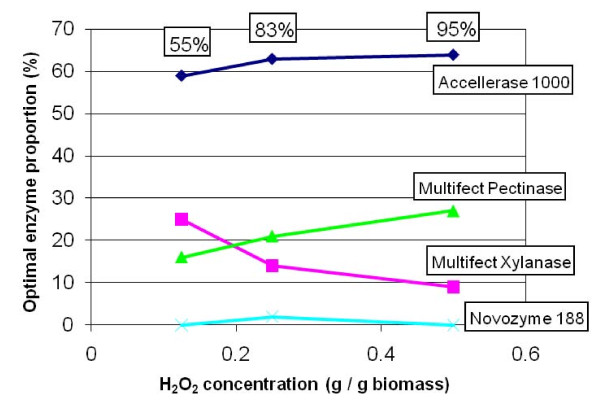
**Optimal proportions of four commercial enzymes as a function of H_2_O_2 _loading during alkaline hydrogen peroxide (AHP) pretreatment**. The data are from Table 1. Boxed percentages across the top of the graph are the glucose (Glc) yields at each H_2_O_2 _loading, expressed as a percentage of the total measured monomeric Glc in the biomass.

A trend in relative proportions of the four enzymes when going from low to high H_2_O_2 _loadings was the decreasing importance of Multifect Xylanase and the increasing importance of Multifect Pectinase (Table 1, Figure 5). A possible explanation for this is that more severe pretreatments (that is, at higher H_2_O_2 _loading) are more effective at dissociating xylan from cellulose, and thus less xylanase is required to facilitate access of the cellulases to the cellulose [31,35]. This hypothesis assumes that it is actually the endo-β1,4-xylanase activity in Multifect Xylanase that contributes to Glc release. Why Multifect Pectinase showed the opposite trend of becoming more important at higher H_2_O_2 _loading is less clear. This enzyme preparation contains more than 130 proteins and is particularly rich in enzymes active on hemicelluloses and pectins, many of which could potentially contribute to Glc yield (JS Scott-Craig and JD Walton, unpublished results).

A mixture of the four commercial enzymes was also optimized for AFEX-pretreated corn stover (Table 1, Figure 4). With the optimized mixture, the yield of Glc was somewhat higher from AFEX-corn stover than from stover pretreated with an H_2_O_2 _loading of 0.125 g/g (61.5 vs 55.0%). However, AFEX was much less effective than either 0.25 or 0.5 g H_2_O_2_/g biomass (61.5% vs 83.0% or 95.0%, respectively) (Table 1).

There were striking differences between the optimal proportions of the four commercial enzyme preparations for AHP-stover compared to AFEX-stover. The optimal proportion of Accellerase 1000 was approximately 60% to 65% for both, but a higher proportion of Multifect Xylanase was needed for release of Glc from AFEX-stover than from stover subjected to any of the AHP conditions (Table 1, Figure 4). That is, for AFEX-stover, the optimal proportion of Multifect Xylanase was 34%, whereas the optimal proportion for AHP-stover ranged from 9% to 25% (Table 1). In place of Multifect Xylanase, Multifect Pectinase was relatively more important for Glc release from AHP-stover at all H_2_O_2 _loadings (optima ranging from 16% to 25%) but was not required at all for Glc release from AFEX-stover (that is, an optimum of 0%) (Table 1, Figure 4). At this point, it is difficult to speculate about the reasons for these differences between AFEX and AHP, mostly because of the poorly defined complexity of these commercial enzyme preparations.

**Figure 6:**
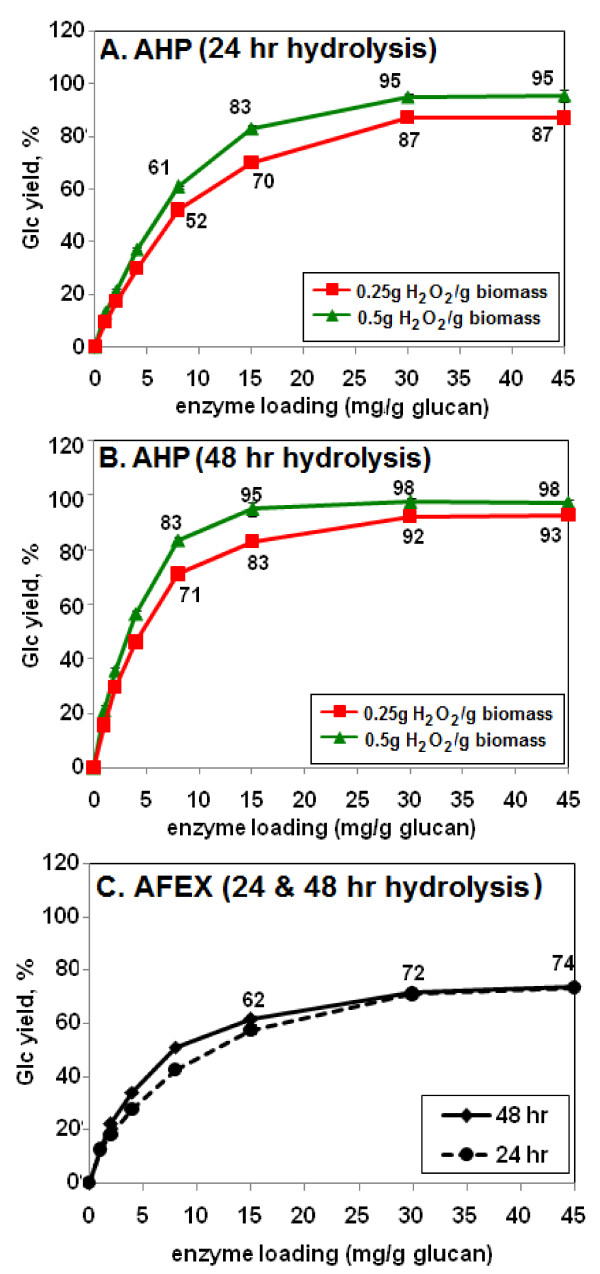
**Glucose (Glc) yield as a function of enzyme loading for different pretreatment conditions, H**_**2**_**O**_**2 **_**loadings, and enzyme hydrolysis times**. The enzymes used for the hydrolysis were mixtures of four commercial enzymes as established for each specific pretreatment condition (that is, H_2_O_2 _loading) at an enzyme loading of 15 mg/g glucan and 48 h hydrolysis (see Table 1). **(a) **Alkaline hydrogen peroxide (AHP) pretreatment and 24 h enzyme hydrolysis. **(b) **AHP pretreatment and 48 h digestion. **(c) **Ammonia fiber expansion (AFEX) pretreatment, 24 h and 48 h enzyme hydrolysis. Biomass loading during the AHP pretreatment was 10%. For AFEX conditions see Methods. All biomass loadings in the enzyme hydrolysis step were 0.2% glucan. Glc yield values indicate yield as a percentage of total measured monomeric Glc content of the biomass. Numbers next to the data points are the Glc yields (y axis values).

The effect of varying the enzyme loading on monomeric Glc yields was examined with the four-enzyme cocktails optimized for the conditions shown in Table 1 (that is, 15 mg enzyme/g glucan and 48 h hydrolysis). Digestions were performed for 24 h or 48 h. In 24 h, stover pretreated with 0.5 g H_2_O_2_/g biomass at 10% loading released 83% of available Glc at an enzyme loading of 15 mg/g glucan (Figure 6a) compared to 71% with Accellerase 1000 alone (Figure 2c). In 48 h, 95% of the available Glc was released with an optimized enzyme mixture at 15 mg/g glucan (Figure 6b), compared to 82% with Accellerase 1000 alone (Figure 2d). When analyzed in parallel, the commercial enzyme mixture optimized for AFEX pretreatment (Table 1) gave 60% or 62% of maximum Glc release in 24 h or 48 h, respectively, and did not exceed 74% even at the highest enzyme loading of 45 mg/g glucan (Figure 6c). With less severe AFEX conditions, as specified in Banerjee *et al*. [20], the same optimized commercial mixture gave a Glc yield of 60.3 ± 1.8% in 48 h.

### Synthetic mixtures optimized for AHP

**Figure 7:**
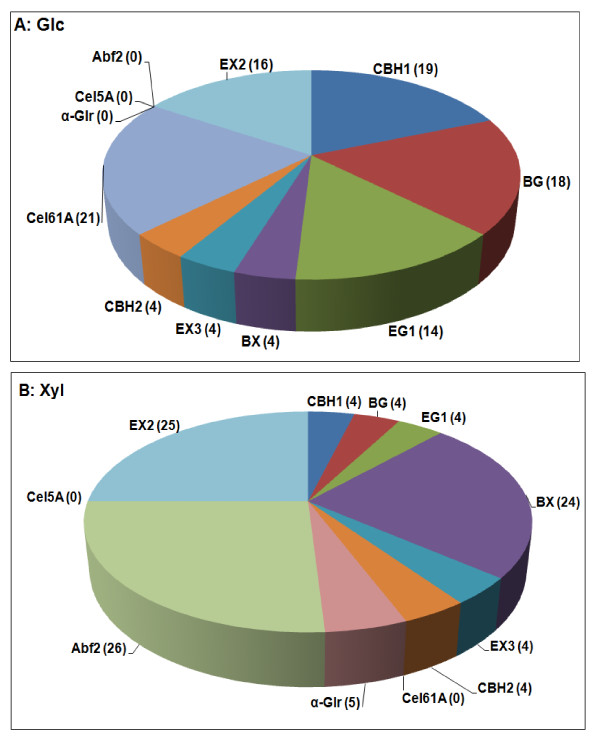
**Optimization of an 11-component synthetic enzyme mixture for (a) glucose (Glc) or (b) xylose (Xyl) yields from alkaline hydrogen peroxide (AHP)-corn stover**. AHP conditions were 10% biomass loading, 0.5 g H_2_O_2_/g biomass, 24 h pretreatment, and 48 h enzymatic hydrolysis. Raw data and statistical analysis are shown in Additional file 1, Tables S9 and S10.

Synthetic mixtures of pure enzymes can reveal the relative importance of specific enzymes in a way that mixtures of incompletely defined commercial enzymes cannot [31,32]. We showed that an optimized synthetic mixture containing 11 components released 52% and 41% of available Glc and Xyl, respectively, from AFEX-stover, and 69% and 55% of available Glc and Xyl, respectively, from stover treated with AHP at 2% biomass loading and 0.5 g H_2_O_2_/g biomass [20]. Using GENPLAT, we determined the optimal proportions of the same 11 enzymes for hydrolysis of AHP-stover pretreated at 10% biomass loading and the same H_2_O_2 _loading of 0.5 g/g (Figure 7). The optimized mixture released 84.6 ± 1.3% of available Glc and 64.7 ± 2.6% of available Xyl. That is, the synthetic mixture showed the same trend seen with Accellerase 1000 alone or an optimized four-component commercial mixture of giving higher Glc yields at higher biomass loading (Figure 1 and Table 1). The relevant raw data are shown in Additional file 1, Table S9 and the statistics for the 11-component model in Additional file 1, Table S10.

We earlier optimized a 16-component mixture for AHP-corn stover pretreated at 2% biomass loading and a loading of 0.5 g H_2_O_2_/g biomass [20]. In the current experiment, we optimized an 11-component mixture. Because in both the earlier [20] and the current (Figure 7) experiments only eight enzymes influenced Glc yield (that is, the others had an optimal proportion of 0%), the two sets of results are comparable. Although the only difference between the earlier and current experiments was the use of a higher biomass loading (10% vs 2%), the optimal proportions of some of the enzymes changed rather dramatically. In particular, the optimal proportion of CBH1 decreased from 31% to 19%, and the optimal proportion of CBH2 decreased from 12% to 4%. That is, the total CBH proportion declined from 43% to 23%. In contrast, the optimal proportion of endoglucanase (EG1) increased from 8% to 14%, and the optimum proportion of β-glucosidase (BG) increased from 5% to 18%. One possible explanation for these observations is that AHP reduces cellulose crystallinity or otherwise renders the cellulose more susceptible to hydrolysis by the enzymes that are responsible for disrupting the cellulose crystallinity, namely, CBH1 and CBH2, making these two enzymes proportionally less important than EG1 and BG [17,36]. By this hypothesis, higher biomass loadings are effectively increasing the severity of the AHP pretreatment (due to the increased molar concentration of H_2_O_2 _and NaOH), leading to progressively greater cellulose susceptibility to CBH. As this occurs, the limiting factor in the pathway of conversion of cellulose to Glc shifts from decrystallization (catalyzed by CBH1 and CBH2) to hydrolysis of non-crystalline glucan (catalyzed by EG and BG). Insofar as action on crystalline cellulose is the generally rate-limiting step in overall cellulose hydrolysis, this hypothesis is consistent with the increased Glc yields observed at higher biomass loadings.

An alternative reason for why a higher proportion of BG is needed at 10% pretreatment loading compared to 2% might be a result of the higher Glc yields seen at the higher loading. As the rate of cellulose hydrolysis by CBH1, CBH2, and EG1 increases, cellobiose concentrations would rise, leading to more feedback inhibition by cellobiose. At a total glucan concentration of 2 mg/ml and with 87% conversion of glucan to cellobiose, the cellobiose concentration in our samples could attain a theoretical maximum concentration of 4.8 mM, which is within the range known to inhibit CBH1 and possibly also EG1 [37]. Therefore, as the cellobiose concentration in the reaction mixture approaches inhibitory levels, BG would be predicted to become more important for optimal Glc yields, as was observed (Figure 7).

### Further improvements of AHP

Several factors were examined that might further improve the efficiency of AHP. These were pH stabilization, extended residence time, and yet higher biomass loadings.

**Figure 8:**
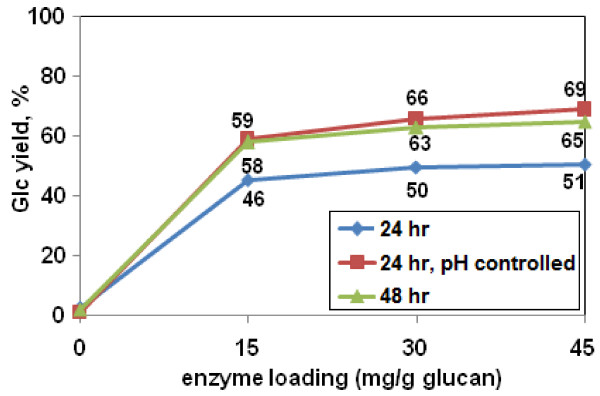
**Improvement in glucose (Glc) yield with pH adjustment or extended residence time**. Biomass loading was 10%, H_2_O_2 _loading was 0.125 g/g biomass, and enzyme digestion was Accellerase 1000 for 48 h. Varied conditions were time of pretreatment (24 h or 48 h), and with or without periodic pH readjustment to 11.5 ('pH controlled') during the pretreatment. Glc yield values indicate yield as a percentage of total measured monomeric Glc content of the biomass. Numbers next to the data points are the Glc yields (y axis values).

An H_2_O_2 _loading of 0.125 g/g biomass was less effective than either 0.25 or 0.5 g H_2_O_2_/g biomass (Figure 3). However, at an H_2_O_2 _loading of 0.125 g/g biomass, the pH tended to drift downward from the optimum of 11.5 over the course of a 24 h pretreatment. This pH drift did not occur at 0.25 or 0.5 g H_2_O_2_/g biomass. Therefore, we tested whether stabilizing the pH at 11.5 would improve the effectiveness of AHP pretreatment at lower H_2_O_2 _loadings. When the pH of an AHP pretreatment at 10% biomass loading and 0.125 g H_2_O_2_/g biomass was held constant by manually readjusting the pH back to 11.5 every 6 h with 5 M NaOH, subsequent yields of monomeric Glc improved from 46% to 59% (using Accellerase 1000 at 15 mg/g glucan) (Figure 8). With a four-component enzyme mixture optimized specifically for this pretreatment condition, the Glc yields could be further increased to 73.8% (Table 2).

**Table 2:** Optimization of mixtures of four commercial enzymes for different alkaline hydrogen peroxide (AHP) pretreatments

Pretreatment conditions	Optimal enzyme proportions (%)				Glc yield (%)	
	
	Acc 1000	Multifect xylanase	Multifect pectinase	Novozyme 188	MP	Exptl
0.125 g H_2_O_2_/g biomass (10% biomass loading, 24 h, controlled pH)	63	24	13	0	72	73.8 ± 2.2

0.125 g H_2_O_2_/g biomass (10% biomass loading, 48 h, no pH control)	63	14	20	3	66	66.3 ± 1.3

0.5 g H_2_O_2_/g biomass (15% biomass loading, 24 h, no pH control)	63	10	27	0	100	96.1 ± 2.1

0.5 g H_2_O_2_/g biomass (20% biomass loading, 24 h, no pH control)	63	10	27	0	100	96.8 ± 1.0

0.125 g H_2_O_2_/g biomass (15% biomass loading, 48 h, controlled pH)	62	24	15	0	76	76.8 ± 2.2

All models were optimized for glucose (Glc). Glc values indicate yield as a percentage of total measured Glc content of the biomass. Enzyme loading was 15 mg/g glucan. Raw data and statistical analysis are shown in Additional file 1, Tables S11-S16.

Acc 1000 = Accellerase 1000; Exptl = experimental results (mean ± 1 SD, n = 8); MP = model prediction.

The effect of extended residence time was also reinvestigated. Gould [15,29] reported that AHP pretreatment of 2% biomass with 0.5 g H_2_O_2_/g biomass was essentially complete at 8 h. At a biomass loading of approximately 8.6% (w/v) and an H_2_O_2 _loading of approximately 0.25 g/g biomass, Saha and Cotta [22] found that AHP pretreatment was essentially complete at 6 h. However, neither of these studies examined the effect of extended residence time at low H_2_O_2 _loadings. In fact, extending the residence time from 24 h to 48 h at a low H_2_O_2 _loading (0.125 g/g biomass), with no pH adjustment, caused an increase in Glc yields in response to Accellerase 1000 from 46% to 58% (Figure 8). With a four-component enzyme mixture optimized specifically for this pretreatment condition, Glc yields could be increased further to 66.3% (Table 2). In summary, with Accellerase 1000 alone, pH adjustment or extended residence time caused a similar enhancement of monomeric Glc yields (that is, from 46% up to 58% to 59%), but when using an optimized commercial cocktail, pH adjustment caused a bigger enhancement than increased pretreatment time (that is, from 66% to 74%) (Figure 8 and Table 2).

**Figure 9:**
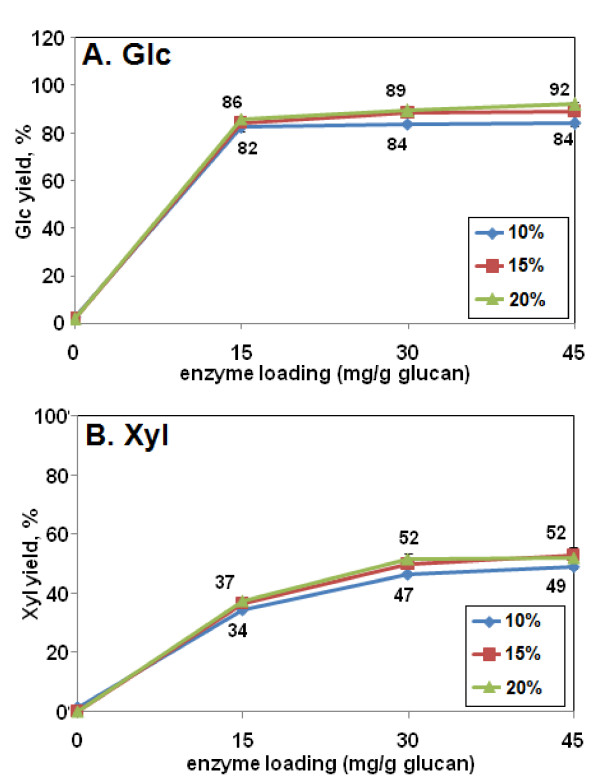
**Effect of high biomass loadings on (a) glucose (Glc) and (b) xylose (Xyl) yield from alkaline hydrogen peroxide (AHP)-pretreated corn stover**. The H_2_O_2 _loading in the AHP pretreatment was 0.5 g/g biomass and the residence time was 24 h. The enzyme digestion was 48 h with Accellerase 1000. Glc and Xyl yields values indicate yield as a percentage of total measured monomeric Glc and Xyl content of the biomass.

In regard to biomass loading during pretreatment, the earlier results indicated that 10% loading was superior to 2%, 6%, or 8% (Figures 1 and 2). Therefore, even higher loadings were tested. At loadings of 15% or 20%, with subsequent digestion with Accellerase 1000 alone, sugar yields improved modestly, by 4% to 8% for Glc and 3% to 5% for Xyl (Figure 9).

Finally, several of these enhanced conditions, that is, high biomass loading (15%), low H_2_O_2 _loading (0.125 g/g biomass), extended residence time (48 h), pH adjustment, and optimized enzyme mixtures, were tested together (Table 2). With an optimized four-component mixture of commercial enzymes, a monomeric Glc yield of 76.8% was attained (Table 2).

## Discussion

Our results confirm that AHP is an effective pretreatment of corn stover for subsequent enzymatic saccharification to monomeric Glc and Xyl, and that it can be further improved in performance and economics by increasing the biomass loading, reducing the H_2_O_2 _loading, extending the pretreatment time, and maintaining the pH.

The advantages of AHP over other existing effective pretreatments are several. First, it uses readily available and environmentally benign chemicals, namely, hydrogen peroxide and sodium hydroxide. Second, it operates at low temperature (21°C to 50°C) and atmospheric pressure, so no expensive specialized reactors are necessary. It can be performed in any standard scientific laboratory, which means that researchers interested in studying AHP do not have to rely on outside collaborators with specialized equipment. Third, it is less expensive than two other highly effective pretreatments, ionic liquids and phosphoric acid/organic solvent. Phosphoric acid/ethanol was reported to give a monomeric Glc yield of > 89% with a very low enzyme loading (0.66 mg cellulase/g glucan), but it requires a phosphoric acid:biomass loading of approximately 13:1 (w/w) [6,7]. Ionic liquids gave 96% digestibility of switchgrass but use a reagent: biomass ratio of 32:1 [8]. Practical implementation of these two effective pretreatments will require highly efficient recycling of the respective reagents.

The main potential disadvantages of AHP pretreatment are the cost of H_2_O_2 _and the presence of significant residual salt. At an H_2_O_2 _price of US$0.80 to $1.00/kg (on a 100% basis) (Dr Philip Block, FMC Corporation, personal communication), the current cost for processing 1 metric ton of biomass would be US$102 to US$125 at an H_2_O_2 _loading of 0.125 g/g biomass, plus US$36 for NaOH (at $300/ton). However, AHP can probably be improved to operate at even lower H_2_O_2 _loadings, for example, by H_2_O_2 _stabilization, improved pH control, or H_2_O_2 _recycling [15]. The use of lower H_2_O_2 _loadings would also have the advantage of reducing the consumption of NaOH and acid. In addition, it might be possible to reduce the intrinsic cost of H_2_O_2 _by on-site production or by the development of new methods of production [38].

In these and earlier studies, we used an enzyme loading of 15 mg total protein/g glucan as our standard hydrolysis condition. Comparison of this enzyme loading to other studies in which enzyme loadings are expressed in filter paper units (FPU) is difficult because FPU is a measure of cellulase activity only and does not take into account the contribution of the many other enzymes in most cellulase preparations, such as BG and xylanases, to the release of monomeric Glc and Xyl from lignocellulose [31]. However, a point of comparison can be made to Spezyme CP, a widely used commercial cellulase cocktail. It has been reported to contain between 0.3 and 0.48 FPU/mg protein [35,39], and therefore our standard loading of 15 mg/g glucan corresponds to a Spezyme CP loading of between 4.5 and 7.2 FPU/g glucan.

In the current experiments, the biomass was not washed or otherwise separated into multiple processing streams after pretreatment. Many pretreatment protocols involve post-washing the biomass to remove inhibitors and salts. Depending on the type and severity, some pretreatments also remove some of the lignin and hemicelluloses [8,19,23,24,26-28]. However, washing uses a large amount of water (exactly how much is rarely indicated), and, depending on the severity of the pretreatment, removes a proportion of the cellulose and hemicellulose. This lowers the energy content of the biomass and, for laboratory-scale studies, necessitates reanalysis of the glucan and xylan content post pretreatment in order to calculate final sugar yields [19]. In our experiments, all of the original material was still present regardless of the pretreatment severity. Since no cellulose or hemicellulose was lost, reanalysis of the biomass composition was unnecessary. The main disadvantage of not washing after AHP is that the final product contains salt from the NaOH and HCl. With AHP conditions of 2% biomass loading and 0.5 g H_2_O_2_/g biomass, the final NaCl concentration was approximately 200 mM (Additional file 1, Table S1). At the highest biomass loading (20%) and highest H_2_O_2 _loading, the NaCl concentration was approximately 1.5 M (Additional file 1, Table S1). This high salt concentration was not a problem in our experiments because all enzyme digestions were performed at 0.2% glucan loading, which lowered the salt concentration to approximately 20 mM, but it might become a problem if enzymatic hydrolysis (and fermentation) were performed at industrially relevant high-solids loading.

In addition to lowering the consumption of H_2_O_2_, reducing the H_2_O_2 _loading results in less residual salt in the pretreated material. For example, at 10% biomass loading and 0.125 g H_2_O_2_/g biomass, the NaCl concentration in the biomass after neutralization was approximately 350 mM (Additional file 1, Table S1). Saha and Cotta [21,22] used AHP-pretreatment on barley and wheat straw at approximately 10% biomass loading and approximately 2.5% H_2_O_2 _and successfully digested and fermented the resulting material without post-washing. Residual salt did, however, inhibit butanol fermentation by *Clostridum beijerinckii *[40]. Possible routes to alleviating the problem of salt from AHP include further decreasing the H_2_O_2 _loading, removing the salt after pretreatment, or performing the fermentation with salt-tolerant organisms. *Saccharomyces cerevisiae *strains able to tolerate NaCl concentrations of greater than 0.5 M NaCl have been developed under laboratory conditions [41-43].

Previous studies with AHP have shown that it solubilizes lignin but its effect on cellulose crystallinity is unclear [17,19,36]. One or both of these modifications could be contributing to the effectiveness of AHP as a pretreatment. Additional studies on the mechanisms of AHP are needed, for example, on the relative composition of the insoluble and soluble fractions after pretreatment. In the experiments reported here, the soluble and insoluble portions of the biomass were not separated after pretreatment, and therefore the total (free, oligomeric, and polymeric) Glc and Xyl composition of the biomass did not change between the original biomass and the hydrolyzed material.

AHP satisfies many criteria of an ideal pretreatment, in particular high yields of fermentable sugars at low enzyme loadings, low cost of pretreatment reactors, minimal post-pretreatment conditioning, low disposal challenges, and low heat and power demands [4]. Further improvements will be necessary, however, before AHP can be considered an economically relevant pretreatment, in particular a reduction in the cost of hydrogen peroxide.

## Conclusions

Corn stover pretreated by AHP gives high monomeric Glc and Xyl yields at low enzyme loadings. AHP appear to be amenable to further improvements that could lead to its development as an economically viable step in lignocellulose to ethanol conversion.

## Competing interests

The authors declare that they have no competing interests.

## Authors' contributions

GB and SC performed the alkaline peroxide pretreatments, designed and executed the digestion experiments, performed the statistical analyses and helped write the paper; JSC cloned genes and expressed proteins; DBH made conceptual contributions and helped write the paper; JDW contributed to experimental design and drafted the final manuscript. All authors read and approved the final manuscript.

## Supplementary Material

Additional file 1 **Supplementary supporting data**. Table S1. Biomass and reagent loadings for experiments described in the paper. Table S2. Amounts of NaOH added during pH adjustment experiments. Table S3. Compensation calculations for NaCl (as NaOH plus HCl) added during pretreatment and pH neutralization. Table S4. Experimental results for glucose (Glc) optimization obtained from digestion of alkaline hydrogen peroxide (AHP)-treated corn stover (0.125 g H_2_O_2_/g biomass, 10% solids loading). Table S5. Experimental results for Glc optimization obtained from digestion of AHP-treated corn stover (0.25 g H_2_O_2_/g biomass, 10% solids loading). Table S6. Experimental results for Glc optimization obtained from digestion of AHP-treated corn stover (0.5 g H_2_O_2_/g biomass, 10% solids loading). Table S7. Experimental results for Glc and xylose (Xyl) optimization obtained from digestion of ammonia fiber expansion (AFEX)-treated corn stover with mixtures of four commercial enzyme preparations. Table S8. Statistical analysis for Glc and Xyl optimization from AHP and AFEX-treated corn stover. Table S9. Experimental results for Glc and Xyl optimization obtained from digestion of AHP-treated corn stover (0.5 g H_2_O_2_/g biomass) with 11-component synthetic enzyme mixture. Table S10. Statistical analysis of the 11-component optimization experiment. Table S11. Experimental results for Glc optimization obtained from digestion of AHP-treated corn stover (0.125 g H_2_O_2_/g biomass, 10% solids loading with controlled pH for 24 h). Table S12. Experimental results for Glc optimization obtained from digestion of AHP-treated corn stover (0.125 g H_2_O_2_/g biomass, 10% solids loading for 48 h and no pH control). Table S13. Experimental results for Glc optimization obtained from digestion of AHP-treated corn stover (0.5 g H_2_O_2_/g biomass, 15% solids loading, 24 h, no pH control). Table S14. Experimental results for Glc optimization obtained from digestion of AHP-treated corn stover (0.5 g H_2_O_2_/g biomass, 20% solids loading, 24 h, no pH control). Table S15. Experimental results for Glc optimization obtained from digestion of AHP-treated corn stover (0.125 g H_2_O_2_/g biomass, 15% solids loading, 48 h, controlled pH). Table S16. Statistical analysis for Glc optimization from corn stover under different AHP conditions. Click here for file
