# Supplementary material for: Alkaline peroxide pretreatment of corn stover: effects of biomass, peroxide, and enzyme loading and composition on yields of glucose and xylose
Source: Biotechnol Biofuels. 2011 Jun 9;4:16. doi: 10.1186/1754-6834-4-16 (PMC3123552; doi:10.1186/1754-6834-4-16)
Supplement: Additional file 1 — Supplementary supporting data. Table S1. Biomass and reagent loadings for experiments described in the paper. Table S2. Amounts of NaOH added during pH adjustment experiments. Table S3. Compensation calculations for NaCl (as NaOH plus HCl) added during pretreatment and pH neutralization. Table S4. Experimental results for glucose (Glc) optimization obtained from digestion of alkaline hydrogen peroxide (AHP)-treated corn stover (0.125 g H2O2/g biomass, 10% solids loading). Table S5. Experimental results for Glc optimization obtained from digestion of AHP-treated corn stover (0.25 g H2O2/g biomass, 10% solids loading). Table S6. Experimental results for Glc optimization obtained from digestion of AHP-treated corn stover (0.5 g H2O2/g biomass, 10% solids loading). Table S7. Experimental results for Glc and xylose (Xyl) optimization obtained from digestion of ammonia fiber expansion (AFEX)-treated corn stover with mixtures of four commercial enzyme preparations. Table S8. Statistical analysis for Glc and Xyl optimization from AHP and AFEX-treated corn stover. Table S9. Experimental results for Glc and Xyl optimization obtained from digestion of AHP-treated corn stover (0.5 g H2O2/g biomass) with 11-component synthetic enzyme mixture. Table S10. Statistical analysis of the 11-component optimization experiment. Table S11. Experimental results for Glc optimization obtained from digestion of AHP-treated corn stover (0.125 g H2O2/g biomass, 10% solids loading with controlled pH for 24 h). Table S12. Experimental results for Glc optimization obtained from digestion of AHP-treated corn stover (0.125 g H2O2/g biomass, 10% solids loading for 48 h and no pH control). Table S13. Experimental results for Glc optimization obtained from digestion of AHP-treated corn stover (0.5 g H2O2/g biomass, 15% solids loading, 24 h, no pH control). Table S14. Experimental results for Glc optimization obtained from digestion of AHP-treated corn stover (0.5 g H2O2/g biomass, 20% solids loading, 24 [file 1754-6834-4-16-S1.DOC]

March 26, 2011

**Supplementary Supporting Data**

G. Banerjee et al. **Alkaline peroxide pretreatment of corn stover: effects of biomass loading, peroxide concentration, and enzyme concentration and composition on yields of glucose and xylose.**

**Table S1** Biomass and reagent loadings for experiments described in the paper. All pretreatment residence times were 24 h except “pH 48”. “pH 24” and “pH 48” indicate pretreatments of 24 h or 48 h, respectively, with periodic pH re-adjustment to pH 11.5 (see Table S3).

| **Biomass (g)** | **Nominal biomass loading (%)** | **30% H_2_O_2_ (ml)** | **Nominal [H_2_O_2_] (g/g biomass)** | **5 M NaOH (ml)** | **Water (ml)** | **Final liquid volume (ml)** | **Final [NaOH] (M)** |
| --- | --- | --- | --- | --- | --- | --- | --- |
| 1 | 2 | 1.66 | 0.5 | 2.0 | 46.6 | 50.3 | 0.2 |
| 1 | 6 | 1.66 | 0.5 | 2.0 | 13.0 | 16.7 | 0.6 |
| 1 | 8 | 1.66 | 0.5 | 2.0 | 8.8 | 12.5 | 0.8 |
| 1 | 10 | 1.66 | 0.5 | 2.0 | 6.3 | 10.0 | 1.0 |
| 1 | 10 | 0.83 | 0.25 | 1.0 | 8.2 | 10.0 | 0.5 |
| 1 | 10 | 0.42 | 0.125 | 0.5 | 9.1 | 10.0 | 0.25 |
| 1 | 10 | 0.42 | 0.125 (pH 24) | 0.64 | 9.1 | 10.1 | 0.32 |
| 1 | 10 | 0.42 | 0.125 (pH 48) | 0.72 | 9.1 | 10.2 | 0.35 |
| 1 | 15 | 1.66 | 0.5 | 2.0 | 3.0 | 6.7 | 1.5 |
| 1 | 20 | 1.66 | 0.5 | 2.0 | 1.3 | 5.0 | 2.0 |
| 1 | 15 | 0.42 | 0.125 (pH 48) | 0.5 | 5.8 | 6.7 | 0.37 |

**Table S2** Quantities of NaOH added during pH adjustment experiments at 0.125 g H_2_O_2_/g biomass. “pH” indicates the measured pH at each time point before pH adjustment. “NaOH added” indicates amount of 5 M NaOH added to return the pH to 11.5. For 24-hr experiments, the last pH adjustment was at 18 hr; for 48-hr experiments, the last pH adjustment was at 42 hr.

Starting liquid volume was 10 ml.

| **Time point (h)** | **pH** | **NaOH added (μl of 5 M)** |
| --- | --- | --- |
| 0 | 10.92 | 45 |
| 6 | 11.01 | 45 |
| 12 | 11.11 | 25 |
| 18 | 11.26 | 20 |
| 24 | 11.28 | 20 |
| 30 | 11.20 | 20 |
| 36 | 11.25 | 20 |
| 42 | 11.30 | 20 |

**Table S3** Calculations for NaCl concentrations in biomass after pretreatment and neutralization (added as NaOH plus HCl).

| **Biomass added (g)** | **[H_2_O_2_] (g/g biomass) and experimental condtions** | **[NaOH] (g/g biomass)** | **[NaCl] (g/g biomass)** | **Mass of biomass + salt (g)** |
| --- | --- | --- | --- | --- |
| 1 | 0.5 (24 h) | 0.4 | 0.58 | 1.58 |
| 1 | 0.25 (24 h) | 0.2 | 0.29 | 1.29 |
| 1 | 0.125 (24 h) | 0.1 | 0.15 | 1.15 |
| 1 | 0.125 (controlled pH, 24 h) | 0.1 | 0.16 | 1.16 |
| 1 | 0.125 (controlled pH, 48 h) | 0.1 | 0.21 | 1.21 |

**Table S4:** Experimental results for Glc optimization obtained from digestion of AHP-treated corn stover (0.125g H_2_O_2_/g biomass, 10% solids loading) with mixtures of four commercial enzyme preparations. All enzyme loadings were 15 mg protein/g glucan. See Table 1.

| Enzyme proportions | | | | Glc yield, % |
| --- | --- | --- | --- | --- |
| Accellerase 1000 | Multifect- Xylanase | Multifect-Pectinase | Novozyme 188 |
| 1.00 | 0.00 | 0.00 | 0.00 | 40.7 ± 0.4 |
| 0.00 | 1.00 | 0.00 | 0.00 | 7.3 ± 0.3 |
| 0.00 | 0.00 | 1.00 | 0.00 | 12.6 ± 0.4 |
| 0.00 | 0.00 | 0.00 | 1.00 | 8.0 ± 0.4 |
| 0.50 | 0.50 | 0.00 | 0.00 | 46.8 ± 0.2 |
| 0.50 | 0.00 | 0.50 | 0.00 | 46.5 ± 0.5 |
| 0.50 | 0.00 | 0.00 | 0.50 | 43.5 ± 0.7 |
| 0.00 | 0.50 | 0.50 | 0.00 | 14.5 ± 0.7 |
| 0.00 | 0.50 | 0.00 | 0.50 | 10.0 ± 0.3 |
| 0.00 | 0.00 | 0.50 | 0.50 | 11.4 ± 0.3 |
| 0.63 | 0.13 | 0.13 | 0.13 | 48.9 ± 0.5 |
| 0.13 | 0.63 | 0.13 | 0.13 | 41.9 ± 0.6 |
| 0.13 | 0.13 | 0.63 | 0.13 | 39.1 ± 0.5 |
| 0.13 | 0.13 | 0.13 | 0.63 | 39.2 ± 0.5 |
| 0.25 | 0.25 | 0.25 | 0.25 | 46.3 ± 0.5 |

**Table S5:** Experimental results for Glc optimization obtained from digestion of AHP-treated corn stover (0.25g H_2_O_2_/g biomass, 10% solids loading) with mixtures of four commercial enzyme preparations. All enzyme loadings were 15 mg protein/g glucan. See Table 1.

| Enzyme proportions | | | | Glc yield, % |
| --- | --- | --- | --- | --- |
| Accellerase 1000 | Multifect- Xylanase | Multifect-Pectinase | Novozyme 188 |
| 1.00 | 0.00 | 0.00 | 0.00 | 72.1 ± 0.9 |
| 0.00 | 1.00 | 0.00 | 0.00 | 6.3 ± 0.0 |
| 0.00 | 0.00 | 1.00 | 0.00 | 14.8 ± 0.0 |
| 0.00 | 0.00 | 0.00 | 1.00 | 7.7 ± 0.0 |
| 0.50 | 0.50 | 0.00 | 0.00 | 71.3 ± 0.1 |
| 0.50 | 0.00 | 0.50 | 0.00 | 76.6 ± 1.5 |
| 0.50 | 0.00 | 0.00 | 0.50 | 71.3 ± 0.1 |
| 0.00 | 0.50 | 0.50 | 0.00 | 16.0 ± 0.1 |
| 0.00 | 0.50 | 0.00 | 0.50 | 9.5 ± 0.0 |
| 0.00 | 0.00 | 0.50 | 0.50 | 12.2 ± 0.1 |
| 0.63 | 0.13 | 0.13 | 0.13 | 79.2 ± 0.2 |
| 0.13 | 0.63 | 0.13 | 0.13 | 61.7 ± 0.3 |
| 0.13 | 0.13 | 0.63 | 0.13 | 57.3 ± 0.1 |
| 0.13 | 0.13 | 0.13 | 0.63 | 54.8 ± 0.1 |
| 0.25 | 0.25 | 0.25 | 0.25 | 72.0 ± 0.5 |

| Enzyme proportions | | | | Glc yield, % | Xyl yield, % |
| --- | --- | --- | --- | --- | --- |
| Accellerase 1000 | Multifect- Xylanase | Multifect-Pectinase | Novozyme 188 |
| 1.00 | 0.00 | 0.00 | 0.00 | 81.0 ± 0.1 | 31.2 ± 0.7 |
| 0.00 | 1.00 | 0.00 | 0.00 | 8.7 ± 0.5 | 48.9 ± 1.0 |
| 0.00 | 0.00 | 1.00 | 0.00 | 18.3 ± 0.0 | 49.5 ± 0.0 |
| 0.00 | 0.00 | 0.00 | 1.00 | 9.3 ± 0.0 | 14.0 ± 0.3 |
| 0.50 | 0.50 | 0.00 | 0.00 | 81.6 ± 0.1 | 56.7 ± 0.3 |
| 0.50 | 0.00 | 0.50 | 0.00 | 89.9 ± 0.3 | 69.1 ± 1.4 |
| 0.50 | 0.00 | 0.00 | 0.50 | 86.8 ± 0.1 | 40.8 ± 0.6 |
| 0.00 | 0.50 | 0.50 | 0.00 | 19.5 ± 0.0 | 60.3 ± 1.3 |
| 0.00 | 0.50 | 0.00 | 0.50 | 11.3 ± 0.0 | 51.3 ± 0.0 |
| 0.00 | 0.00 | 0.50 | 0.50 | 14.6 ± 0.0 | 43.0 ± 0.3 |
| 0.63 | 0.13 | 0.13 | 0.13 | 92.1 ± 0.4 | 67.6 ± 0.2 |
| 0.13 | 0.63 | 0.13 | 0.13 | 61.9 ± 0.0 | 70.2 ± 0.0 |
| 0.13 | 0.13 | 0.63 | 0.13 | 63.8 ± 0.0 | 64.9 ± 0.4 |
| 0.13 | 0.13 | 0.13 | 0.63 | 60.9 ± 0.2 | 64.4 ± 1.0 |
| 0.25 | 0.25 | 0.25 | 0.25 | 80.9 ± 0.0 | 68.8 ± 1.2 |

**Table S6:** Experimental results for Glc optimization obtained from digestion of AHP-treated corn stover (0.5 g H_2_O_2_/g biomass, 10% solids loading) with mixtures of four commercial enzyme preparations. All enzyme loadings were 15 mg protein/g glucan. See Table 1.

**Table S7: Experimental results for Glc and Xyl optimization obtained from digestion of AFEX-treated corn stover with mixtures of four commercial enzyme preparations. All enzyme loadings were 15 mg protein/g glucan. See Table 1.**

| Enzyme proportions | | | | Glc yield, % | Xyl yield, % |
| --- | --- | --- | --- | --- | --- |
| Accellerase 1000 | Multifect- Xylanase | Multifect-Pectinase | Novozyme 188 |
| 1.00 | 0.00 | 0.00 | 0.00 | 51.3 ± 0.3 | 31.0 ± 0.2 |
| 0.00 | 1.00 | 0.00 | 0.00 | 8.4 ± 0.0 | 42.4 ± 0.3 |
| 0.00 | 0.00 | 1.00 | 0.00 | 16.0 ± 0.1 | 40.9 ± 0.5 |
| 0.00 | 0.00 | 0.00 | 1.00 | 7.8 ± 0.0 | 7.9 ± 0.1 |
| 0.50 | 0.50 | 0.00 | 0.00 | 55.1 ± 0.3 | 46.5 ± 0.5 |
| 0.50 | 0.00 | 0.50 | 0.00 | 51.6 ± 1.0 | 54.6 ± 0.1 |
| 0.50 | 0.00 | 0.00 | 0.50 | 48.1 ± 0.4 | 34.3 ± 0.0 |
| 0.00 | 0.50 | 0.50 | 0.00 | 16.9 ± 0.3 | 48.0 ± 0.9 |
| 0.00 | 0.50 | 0.00 | 0.50 | 10.6 ± 0.0 | 42.1 ± 0.5 |
| 0.00 | 0.00 | 0.50 | 0.50 | 13.1 ± 0.3 | 35.6 ± 0.6 |
| 0.63 | 0.13 | 0.13 | 0.13 | 56.7 ± 1.5 | 51.1 ± 0.5 |
| 0.13 | 0.63 | 0.13 | 0.13 | 45.6 ± 0.6 | 51.2 ± 1.1 |
| 0.13 | 0.13 | 0.63 | 0.13 | 40.7 ± 0.4 | 50.6 ± 1.5 |
| 0.13 | 0.13 | 0.13 | 0.63 | 40.9 ± 0.4 | 51.4 ± 1.0 |
| 0.25 | 0.25 | 0.25 | 0.25 | 49.1 ± 0.4 | 51.1 ± 1.2 |

**Table S8:** Statistical analysis for Glc and Xyl optimization from AHP- and AFEX-treated corn stover (see Table 1).

| Pre-treatment conditions | ANOVA for model optimized for | p-value | F-value | R^2 | Adjusted R^2 | Predicted R^2 | Difference between Adj and Pred R^2 | Adequate Precision |
| --- | --- | --- | --- | --- | --- | --- | --- | --- |
| 0.125g H_2_O_2_/g, 10% biomass loading | Glc | <0.0001 | 15.3 | 0.88 | 0.82 | 0.74 | 0.08 | 10.7 |
| 0.25g H_2_O_2_/g, 10% biomass loading | Glc | <0.0001 | 20.0 | 0.90 | 0.86 | 0.78 | 0.08 | 11.7 |
| 0.5g H_2_O_2_/g, 10% biomass loading | Glc | <0.0001 | 45.0 | 0.94 | 0.92 | 0.89 | 0.03 | 17.0 |
|  | Xyl | <0.0001 | 17.0 | 0.89 | 0.84 | 0.76 | 0.08 | 14.4 |
| AFEX | Glc | <0.0001 | 21.5 | 0.91 | 0.87 | 0.80 | 0.07 | 12.5 |
|  | Xyl | <0.0001 | 16.0 | 0.88 | 0.83 | 0.75 | 0.08 | 15.0 |

**Table S9** Experimental results for Glc and Xyl optimization obtained from digestion of AHP-treated corn stover (0.5 g H_2_O_2_/g biomass) with 11-component synthetic enzyme cocktail (see Figure 7). Loading was 15 mg protein/g glucan.

| Sample number | **Enzyme proportions** | | | | | | | | | | | **Glc yield,%** | **Xyl yield,%** |
| --- | --- | --- | --- | --- | --- | --- | --- | --- | --- | --- | --- | --- | --- |
|  | **CBH1** | **BG** | **EG1** | **BXL** | **EX3** | **CBH2** | **Cel61A** | **α-Glr** | **Abf2** | **Cel5A** | **EX2** |  |  |
| 1 | 0.80 | 0.04 | 0.04 | 0.04 | 0.04 | 0.04 | 0.00 | 0.00 | 0.00 | 0.00 | 0.00 | 66.6 ± 0.4 | 43.6 ± 1.7 |
| 2 | 0.04 | 0.80 | 0.04 | 0.04 | 0.04 | 0.04 | 0.00 | 0.00 | 0.00 | 0.00 | 0.00 | 52.5 ± 2.5 | 32.6 ± 1.4 |
| 3 | 0.04 | 0.04 | 0.80 | 0.04 | 0.04 | 0.04 | 0.00 | 0.00 | 0.00 | 0.00 | 0.00 | 45.9 ± 0.0 | 28.1 ± 1.1 |
| 4 | 0.04 | 0.04 | 0.04 | 0.80 | 0.04 | 0.04 | 0.00 | 0.00 | 0.00 | 0.00 | 0.00 | 45.8 ± 2.1 | 39.2 ± 0.6 |
| 5 | 0.04 | 0.04 | 0.04 | 0.04 | 0.80 | 0.04 | 0.00 | 0.00 | 0.00 | 0.00 | 0.00 | 53.5 ± 0.2 | 47.2 ± 0.3 |
| 6 | 0.04 | 0.04 | 0.04 | 0.04 | 0.04 | 0.80 | 0.00 | 0.00 | 0.00 | 0.00 | 0.00 | 39.6 ± 0.2 | 21.8 ± 0.9 |
| 7 | 0.04 | 0.04 | 0.04 | 0.04 | 0.04 | 0.04 | 0.76 | 0.00 | 0.00 | 0.00 | 0.00 | 56.9 ± 0.3 | 32.4 ± 0.0 |
| 8 | 0.04 | 0.04 | 0.04 | 0.04 | 0.04 | 0.04 | 0.00 | 0.76 | 0.00 | 0.00 | 0.00 | 46.0 ± 0.4 | 38.1 ± 1.6 |
| 9 | 0.04 | 0.04 | 0.04 | 0.04 | 0.04 | 0.04 | 0.00 | 0.00 | 0.76 | 0.00 | 0.00 | 42.8 ± 0.6 | 34.1 ± 0.0 |
| 10 | 0.04 | 0.04 | 0.04 | 0.04 | 0.04 | 0.04 | 0.00 | 0.00 | 0.00 | 0.76 | 0.00 | 43.7 ± 0.2 | 28.3 ± 1.4 |
| 11 | 0.04 | 0.04 | 0.04 | 0.04 | 0.04 | 0.04 | 0.00 | 0.00 | 0.00 | 0.00 | 0.76 | 50.5 ± 0.7 | 41.9 ± 1.1 |
| 12 | 0.42 | 0.42 | 0.04 | 0.04 | 0.04 | 0.04 | 0.00 | 0.00 | 0.00 | 0.00 | 0.00 | 69.5 ± 2.3 | 37.9 ± 0.2 |
| 13 | 0.42 | 0.04 | 0.42 | 0.04 | 0.04 | 0.04 | 0.00 | 0.00 | 0.00 | 0.00 | 0.00 | 61.9 ± 1.1 | 33.7 ± 0.3 |
| 14 | 0.42 | 0.04 | 0.04 | 0.42 | 0.04 | 0.04 | 0.00 | 0.00 | 0.00 | 0.00 | 0.00 | 56.8 ± 1.3 | 43.9 ± 2.4 |
| 15 | 0.42 | 0.04 | 0.04 | 0.04 | 0.42 | 0.04 | 0.00 | 0.00 | 0.00 | 0.00 | 0.00 | 61.2 ± 1.0 | 42.4 ± 0.5 |
| 16 | 0.42 | 0.04 | 0.04 | 0.04 | 0.04 | 0.42 | 0.00 | 0.00 | 0.00 | 0.00 | 0.00 | 56.6 ± 0.5 | 31.5 ± 0.1 |
| 17 | 0.42 | 0.04 | 0.04 | 0.04 | 0.04 | 0.04 | 0.38 | 0.00 | 0.00 | 0.00 | 0.00 | 72.7 ± 0.3 | 38.7 ± 0.7 |
| 18 | 0.42 | 0.04 | 0.04 | 0.04 | 0.04 | 0.04 | 0.00 | 0.38 | 0.00 | 0.00 | 0.00 | 59.8 ± 0.3 | 42.5 ± 0.1 |
| 19 | 0.42 | 0.04 | 0.04 | 0.04 | 0.04 | 0.04 | 0.00 | 0.00 | 0.38 | 0.00 | 0.00 | 60.5 ± 0.2 | 44.5 ± 1.0 |
| 20 | 0.42 | 0.04 | 0.04 | 0.04 | 0.04 | 0.04 | 0.00 | 0.00 | 0.00 | 0.38 | 0.00 | 62.5 ± 0.2 | 34.2 ± 1.2 |
| 21 | 0.42 | 0.04 | 0.04 | 0.04 | 0.04 | 0.04 | 0.00 | 0.00 | 0.00 | 0.00 | 0.38 | 70.1 ± 1.5 | 42.3 ± 0.3 |
| 22 | 0.04 | 0.42 | 0.42 | 0.04 | 0.04 | 0.04 | 0.00 | 0.00 | 0.00 | 0.00 | 0.00 | 66.6 ± 0.2 | 38.6 ± 0.5 |
| 23 | 0.04 | 0.42 | 0.04 | 0.42 | 0.04 | 0.04 | 0.00 | 0.00 | 0.00 | 0.00 | 0.00 | 52.7 ± 0.4 | 40.8 ± 1.5 |
| 24 | 0.04 | 0.42 | 0.04 | 0.04 | 0.42 | 0.04 | 0.00 | 0.00 | 0.00 | 0.00 | 0.00 | 58.1 ± 0.7 | 52.5 ± 2.0 |
| 25 | 0.04 | 0.42 | 0.04 | 0.04 | 0.04 | 0.42 | 0.00 | 0.00 | 0.00 | 0.00 | 0.00 | 52.9 ± 0.1 | 28.1 ± 0.1 |
| 26 | 0.04 | 0.42 | 0.04 | 0.04 | 0.04 | 0.04 | 0.38 | 0.00 | 0.00 | 0.00 | 0.00 | 68.3 ± 1.0 | 34.5 ± 0.8 |
| 27 | 0.04 | 0.42 | 0.04 | 0.04 | 0.04 | 0.04 | 0.00 | 0.38 | 0.00 | 0.00 | 0.00 | 48.5 ± 0.4 | 38.5 ± 0.5 |
| 28 | 0.04 | 0.42 | 0.04 | 0.04 | 0.04 | 0.04 | 0.00 | 0.00 | 0.38 | 0.00 | 0.00 | 49.3 ± 1.0 | 42.4 ± 1.6 |
| 29 | 0.04 | 0.42 | 0.04 | 0.04 | 0.04 | 0.04 | 0.00 | 0.00 | 0.00 | 0.38 | 0.00 | 52.1 ± 0.5 | 36.4 ± 0.7 |
| 30 | 0.04 | 0.42 | 0.04 | 0.04 | 0.04 | 0.04 | 0.00 | 0.00 | 0.00 | 0.00 | 0.38 | 67.5 ± 0.1 | 41.2 ± 2.0 |
| 31 | 0.04 | 0.04 | 0.42 | 0.42 | 0.04 | 0.04 | 0.00 | 0.00 | 0.00 | 0.00 | 0.00 | 57.3 ± 0.0 | 50.1 ± 0.8 |
| 32 | 0.04 | 0.04 | 0.42 | 0.04 | 0.42 | 0.04 | 0.00 | 0.00 | 0.00 | 0.00 | 0.00 | 58.8 ± 0.4 | 39.7 ± 0.2 |
| 33 | 0.04 | 0.04 | 0.42 | 0.04 | 0.04 | 0.42 | 0.00 | 0.00 | 0.00 | 0.00 | 0.00 | 56.5 ± 0.1 | 29.1 ± 1.3 |
| 34 | 0.04 | 0.04 | 0.42 | 0.04 | 0.04 | 0.04 | 0.38 | 0.00 | 0.00 | 0.00 | 0.00 | 71.2 ± 0.4 | 33.6 ± 3.1 |
| 35 | 0.04 | 0.04 | 0.42 | 0.04 | 0.04 | 0.04 | 0.00 | 0.38 | 0.00 | 0.00 | 0.00 | 58.3 ± 0.1 | 39.2 ± 0.1 |
| 36 | 0.04 | 0.04 | 0.42 | 0.04 | 0.04 | 0.04 | 0.00 | 0.00 | 0.38 | 0.00 | 0.00 | 56.9 ± 0.2 | 39.5 ± 0.5 |
| 37 | 0.04 | 0.04 | 0.42 | 0.04 | 0.04 | 0.04 | 0.00 | 0.00 | 0.00 | 0.38 | 0.00 | 49.9 ± 0.1 | 29.9 ± 1.6 |
| 38 | 0.04 | 0.04 | 0.42 | 0.04 | 0.04 | 0.04 | 0.00 | 0.00 | 0.00 | 0.00 | 0.38 | 59.3 ± 0.1 | 36.0 ± 0.3 |
| 39 | 0.04 | 0.04 | 0.04 | 0.42 | 0.42 | 0.04 | 0.00 | 0.00 | 0.00 | 0.00 | 0.00 | 50.2 ± 0.2 | 48.8 ± 2.3 |
| 40 | 0.04 | 0.04 | 0.04 | 0.42 | 0.04 | 0.42 | 0.00 | 0.00 | 0.00 | 0.00 | 0.00 | 42.7 ± 0.4 | 43.8 ± 0.5 |
| 41 | 0.04 | 0.04 | 0.04 | 0.42 | 0.04 | 0.04 | 0.38 | 0.00 | 0.00 | 0.00 | 0.00 | 60.9 ± 0.7 | 46.1 ± 0.5 |
| 42 | 0.04 | 0.04 | 0.04 | 0.42 | 0.04 | 0.04 | 0.00 | 0.38 | 0.00 | 0.00 | 0.00 | 46.5 ± 0.3 | 49.2 ± 0.3 |
| 43 | 0.04 | 0.04 | 0.04 | 0.42 | 0.04 | 0.04 | 0.00 | 0.00 | 0.38 | 0.00 | 0.00 | 44.1 ± 0.2 | 54.3 ± 1.9 |
| 44 | 0.04 | 0.04 | 0.04 | 0.42 | 0.04 | 0.04 | 0.00 | 0.00 | 0.00 | 0.38 | 0.00 | 46.6 ± 0.1 | 41.7 ± 2.9 |
| 45 | 0.04 | 0.04 | 0.04 | 0.42 | 0.04 | 0.04 | 0.00 | 0.00 | 0.00 | 0.00 | 0.38 | 62.8 ± 0.3 | 48.0 ± 1.3 |
| 46 | 0.04 | 0.04 | 0.04 | 0.04 | 0.42 | 0.42 | 0.00 | 0.00 | 0.00 | 0.00 | 0.00 | 55.9 ± 0.2 | 35.8 ± 0.9 |
| 47 | 0.04 | 0.04 | 0.04 | 0.04 | 0.42 | 0.04 | 0.38 | 0.00 | 0.00 | 0.00 | 0.00 | 69.4 ± 0.3 | 40.1 ± 2.3 |
| 48 | 0.04 | 0.04 | 0.04 | 0.04 | 0.42 | 0.04 | 0.00 | 0.38 | 0.00 | 0.00 | 0.00 | 52.4 ± 0.1 | 46.0 ± 1.9 |
| 49 | 0.04 | 0.04 | 0.04 | 0.04 | 0.42 | 0.04 | 0.00 | 0.00 | 0.38 | 0.00 | 0.00 | 46.8 ± 0.1 | 43.6 ± 0.2 |
| 50 | 0.04 | 0.04 | 0.04 | 0.04 | 0.42 | 0.04 | 0.00 | 0.00 | 0.00 | 0.38 | 0.00 | 49.7 ± 0.5 | 37.9 ± 0.0 |
| 51 | 0.04 | 0.04 | 0.04 | 0.04 | 0.42 | 0.04 | 0.00 | 0.00 | 0.00 | 0.00 | 0.38 | 58.2 ± 0.0 | 42.4 ± 1.1 |
| 52 | 0.04 | 0.04 | 0.04 | 0.04 | 0.04 | 0.42 | 0.38 | 0.00 | 0.00 | 0.00 | 0.00 | 57.8 ± 0.2 | 30.5 ± 0.6 |
| 53 | 0.04 | 0.04 | 0.04 | 0.04 | 0.04 | 0.42 | 0.00 | 0.38 | 0.00 | 0.00 | 0.00 | 45.3 ± 0.1 | 29.6 ± 0.6 |
| 54 | 0.04 | 0.04 | 0.04 | 0.04 | 0.04 | 0.42 | 0.00 | 0.00 | 0.38 | 0.00 | 0.00 | 44.2 ± 0.1 | 35.3 ± 0.0 |
| 55 | 0.04 | 0.04 | 0.04 | 0.04 | 0.04 | 0.42 | 0.00 | 0.00 | 0.00 | 0.38 | 0.00 | 45.3 ± 0.2 | 25.9 ± 0.4 |
| 56 | 0.04 | 0.04 | 0.04 | 0.04 | 0.04 | 0.42 | 0.00 | 0.00 | 0.00 | 0.00 | 0.38 | 62.5 ± 0.4 | 36.6 ± 0.0 |
| 57 | 0.04 | 0.04 | 0.04 | 0.04 | 0.04 | 0.04 | 0.38 | 0.38 | 0.00 | 0.00 | 0.00 | 60.7 ± 0.1 | 40.8 ± 1.7 |
| 58 | 0.04 | 0.04 | 0.04 | 0.04 | 0.04 | 0.04 | 0.38 | 0.00 | 0.38 | 0.00 | 0.00 | 60.4 ± 0.3 | 39.0 ± 0.4 |
| 59 | 0.04 | 0.04 | 0.04 | 0.04 | 0.04 | 0.04 | 0.38 | 0.00 | 0.00 | 0.38 | 0.00 | 62.8 ± 0.4 | 30.9 ± 1.3 |
| 60 | 0.04 | 0.04 | 0.04 | 0.04 | 0.04 | 0.04 | 0.38 | 0.00 | 0.00 | 0.00 | 0.38 | 73.8 ± 1.0 | 37.3 ± 0.4 |
| 61 | 0.04 | 0.04 | 0.04 | 0.04 | 0.04 | 0.04 | 0.00 | 0.38 | 0.38 | 0.00 | 0.00 | 42.2 ± 0.1 | 43.6 ± 0.2 |
| 62 | 0.04 | 0.04 | 0.04 | 0.04 | 0.04 | 0.04 | 0.00 | 0.38 | 0.00 | 0.38 | 0.00 | 45.0 ± 0.6 | 36.0 ± 1.9 |
| 63 | 0.04 | 0.04 | 0.04 | 0.04 | 0.04 | 0.04 | 0.00 | 0.38 | 0.00 | 0.00 | 0.38 | 59.1 ± 0.1 | 47.6 ± 0.3 |
| 64 | 0.04 | 0.04 | 0.04 | 0.04 | 0.04 | 0.04 | 0.00 | 0.00 | 0.38 | 0.38 | 0.00 | 44.5 ± 0.5 | 37.9 ± 1.8 |
| 65 | 0.04 | 0.04 | 0.04 | 0.04 | 0.04 | 0.04 | 0.00 | 0.00 | 0.38 | 0.00 | 0.38 | 61.4 ± 0.2 | 58.6 ± 1.2 |
| 66 | 0.04 | 0.04 | 0.04 | 0.04 | 0.04 | 0.04 | 0.00 | 0.00 | 0.00 | 0.38 | 0.38 | 68.4 ± 0.3 | 43.1 ± 0.8 |
| 67 | 0.45 | 0.07 | 0.07 | 0.07 | 0.07 | 0.07 | 0.03 | 0.03 | 0.03 | 0.03 | 0.03 | 83.8 ± 1.6 | 71.5 ± 0.6 |
| 68 | 0.07 | 0.45 | 0.07 | 0.07 | 0.07 | 0.07 | 0.03 | 0.03 | 0.03 | 0.03 | 0.03 | 85.4 ± 0.5 | 62.5 ± 0.9 |
| 69 | 0.07 | 0.07 | 0.45 | 0.07 | 0.07 | 0.07 | 0.03 | 0.03 | 0.03 | 0.03 | 0.03 | 82.8 ± 0.4 | 58.9 ± 1.9 |
| 70 | 0.07 | 0.07 | 0.07 | 0.45 | 0.07 | 0.07 | 0.03 | 0.03 | 0.03 | 0.03 | 0.03 | 84.2 ± 0.4 | 72.1 ± 0.6 |
| 71 | 0.07 | 0.07 | 0.07 | 0.07 | 0.45 | 0.07 | 0.03 | 0.03 | 0.03 | 0.03 | 0.03 | 82.0 ± 0.3 | 58.2 ± 1.8 |
| 72 | 0.07 | 0.07 | 0.07 | 0.07 | 0.07 | 0.45 | 0.03 | 0.03 | 0.03 | 0.03 | 0.03 | 81.9 ± 0.1 | 49.9 ± 0.9 |
| 73 | 0.07 | 0.07 | 0.07 | 0.07 | 0.07 | 0.07 | 0.41 | 0.03 | 0.03 | 0.03 | 0.03 | 74.4 ± 0.1 | 55.1 ± 0.2 |
| 74 | 0.07 | 0.07 | 0.07 | 0.07 | 0.07 | 0.07 | 0.03 | 0.41 | 0.03 | 0.03 | 0.03 | 74.5 ± 0.6 | 58.0 ± 0.7 |
| 75 | 0.07 | 0.07 | 0.07 | 0.07 | 0.07 | 0.07 | 0.03 | 0.03 | 0.41 | 0.03 | 0.03 | 73.8 ± 0.1 | 59.3 ± 3.2 |
| 76 | 0.07 | 0.07 | 0.07 | 0.07 | 0.07 | 0.07 | 0.03 | 0.03 | 0.03 | 0.41 | 0.03 | 75.6 ± 0.2 | 58.8 ± 1.8 |
| 77 | 0.07 | 0.07 | 0.07 | 0.07 | 0.07 | 0.07 | 0.03 | 0.03 | 0.03 | 0.03 | 0.41 | 81.4 ± 0.1 | 59.2 ± 0.7 |
| 78 | 0.11 | 0.11 | 0.11 | 0.11 | 0.11 | 0.11 | 0.07 | 0.07 | 0.07 | 0.07 | 0.07 | 81.1 ± 0.8 | 63.2 ± 0.5 |

**Table S10 Statistical analysis of the 11-component optimization experiment** (See Figure 7 and Table S9).

| Model optimized for: | p-value | R^2 | Adjusted R^2 | Predicted R^2 | Difference between Adj and Pred R^2 | Adequate Precision |
| --- | --- | --- | --- | --- | --- | --- |
| Glc (48 hr) | <0.0001 | 0.76 | 0.59 | 0.52 | 0.07 | 8.6 |
| Xyl (48 hr) | <0.0001 | 0.74 | 0.55 | 0.45 | 0.1 | 11.0 |

**Table S11** Experimental results for Glc optimization obtained from digestion of AHP-treated corn stover (0.125 g H_2_O_2_/g biomass, 10% solids loading with controlled pH for 24 hr) with mixtures of four commercial enzyme preparations (see Table 2). All enzyme loadings were 15 mg protein/g glucan.

| Enzyme proportions | | | | Glc yield, % |
| --- | --- | --- | --- | --- |
| Accellerase 1000 | Multifect- Xylanase | Multifect-Pectinase | Novozyme 188 |
| 1.00 | 0.00 | 0.00 | 0.00 | 58.9 ± 0.4 |
| 0.00 | 1.00 | 0.00 | 0.00 | 7.1 ± 0.2 |
| 0.00 | 0.00 | 1.00 | 0.00 | 13.3 ± 0.1 |
| 0.00 | 0.00 | 0.00 | 1.00 | 7.5 ± 0.1 |
| 0.50 | 0.50 | 0.00 | 0.00 | 60.8 ± 0.1 |
| 0.50 | 0.00 | 0.50 | 0.00 | 61.6 ± 0.1 |
| 0.50 | 0.00 | 0.00 | 0.50 | 56.3 ± 0.2 |
| 0.00 | 0.50 | 0.50 | 0.00 | 14.8 ± 0.0 |
| 0.00 | 0.50 | 0.00 | 0.50 | 9.7 ± 0.1 |
| 0.00 | 0.00 | 0.50 | 0.50 | 11.5 ± 0.1 |
| 0.63 | 0.13 | 0.13 | 0.13 | 64.2 ± 0.2 |
| 0.13 | 0.63 | 0.13 | 0.13 | 51.3 ± 0.1 |
| 0.13 | 0.13 | 0.63 | 0.13 | 47.4 ± 0.4 |
| 0.13 | 0.13 | 0.13 | 0.63 | 45.2 ± 0.2 |
| 0.25 | 0.25 | 0.25 | 0.25 | 59.5 ± 0.3 |

**Table S12** Experimental results for Glc optimization obtained from digestion of AHP-treated corn stover (0.125g H_2_O_2_/g biomass, 10% solids loading, 48 hr residence time, no pH control) with mixtures of four commercial enzyme preparations (see Table 2). All enzyme loadings were 15 mg protein/g glucan.

| Enzyme proportions | | | | Glc yield, % |
| --- | --- | --- | --- | --- |
| Accellerase 1000 | Multifect- Xylanase | Multifect-Pectinase | Novozyme 188 |
| 1.00 | 0.00 | 0.00 | 0.00 | 56.3 ± 0.1 |
| 0.00 | 1.00 | 0.00 | 0.00 | 6.8 ± 0.1 |
| 0.00 | 0.00 | 1.00 | 0.00 | 13.1 ± 0.1 |
| 0.00 | 0.00 | 0.00 | 1.00 | 7.3 ± 0.1 |
| 0.50 | 0.50 | 0.00 | 0.00 | 54.6 ± 0.4 |
| 0.50 | 0.00 | 0.50 | 0.00 | 57.9 ± 0.2 |
| 0.50 | 0.00 | 0.00 | 0.50 | 54.2 ± 0.4 |
| 0.00 | 0.50 | 0.50 | 0.00 | 14.6 ± 0.1 |
| 0.00 | 0.50 | 0.00 | 0.50 | 9.1 ± 0.1 |
| 0.00 | 0.00 | 0.50 | 0.50 | 11.3 ± 0.1 |
| 0.63 | 0.13 | 0.13 | 0.13 | 59.5 ± 0.1 |
| 0.13 | 0.63 | 0.13 | 0.13 | 48.9 ± 0.1 |
| 0.13 | 0.13 | 0.63 | 0.13 | 45.2 ± 0.0 |
| 0.13 | 0.13 | 0.13 | 0.63 | 45.6 ± 0.2 |
| 0.25 | 0.25 | 0.25 | 0.25 | 55.4 ± 0.4 |

**Table S13** Experimental results for Glc optimization obtained from digestion of AHP-treated corn stover (0.5g H_2_O_2_/g biomass, 15% solids loading, 24 hr, no pH control) with mixtures of four commercial enzyme preparations (see Table 2). All enzyme loadings were 15 mg protein/g glucan.

| Enzyme proportions | | | | Glc yield, % |
| --- | --- | --- | --- | --- |
| Accellerase 1000 | Multifect- Xylanase | Multifect-Pectinase | Novozyme 188 |
| 1.00 | 0.00 | 0.00 | 0.00 | 86.1 ± 0.6 |
| 0.00 | 1.00 | 0.00 | 0.00 | 9.4 ± 0.2 |
| 0.00 | 0.00 | 1.00 | 0.00 | 21.3 ± 0.5 |
| 0.00 | 0.00 | 0.00 | 1.00 | 9.1 ± 0.1 |
| 0.50 | 0.50 | 0.00 | 0.00 | 83.4 ± 0.4 |
| 0.50 | 0.00 | 0.50 | 0.00 | 93.2 ± 0.0 |
| 0.50 | 0.00 | 0.00 | 0.50 | 90.6 ± 0.1 |
| 0.00 | 0.50 | 0.50 | 0.00 | 23.5 ± 1.6 |
| 0.00 | 0.50 | 0.00 | 0.50 | 12.3 ± 0.3 |
| 0.00 | 0.00 | 0.50 | 0.50 | 17.4 ± 0.6 |
| 0.63 | 0.13 | 0.13 | 0.13 | 90.5 ± 0.3 |
| 0.13 | 0.63 | 0.13 | 0.13 | 74.5 ± 0.0 |
| 0.13 | 0.13 | 0.63 | 0.13 | 74.4 ± 0.3 |
| 0.13 | 0.13 | 0.13 | 0.63 | 68.9 ± 0.1 |
| 0.25 | 0.25 | 0.25 | 0.25 | 89.1 ± 1.1 |

**Table S14: :** Experimental results for Glc optimization obtained from digestion of AHP-treated corn stover (0.5g H_2_O_2_/g biomass, 20% solids loading, 24 h, no pH control) with mixtures of four commercial enzyme preparations (see Table 2). All enzyme loadings were 15 mg protein/g glucan.

| Enzyme proportions | | | | Glc yield, % |
| --- | --- | --- | --- | --- |
| Accellerase 1000 | Multifect- Xylanase | Multifect-Pectinase | Novozyme 188 |
| 1.00 | 0.00 | 0.00 | 0.00 | 85.7 ± 0.1 |
| 0.00 | 1.00 | 0.00 | 0.00 | 10.2 ± 0.1 |
| 0.00 | 0.00 | 1.00 | 0.00 | 24.3 ± 0.1 |
| 0.00 | 0.00 | 0.00 | 1.00 | 8.7 ± 0.0 |
| 0.50 | 0.50 | 0.00 | 0.00 | 86.4 ± 1.6 |
| 0.50 | 0.00 | 0.50 | 0.00 | 92.2 ± 0.7 |
| 0.50 | 0.00 | 0.00 | 0.50 | 87.0 ± 0.8 |
| 0.00 | 0.50 | 0.50 | 0.00 | 24.6 ± 0.0 |
| 0.00 | 0.50 | 0.00 | 0.50 | 12.0 ± 0.2 |
| 0.00 | 0.00 | 0.50 | 0.50 | 18.6 ± 0.6 |
| 0.63 | 0.13 | 0.13 | 0.13 | 92.0 ± 0.8 |
| 0.13 | 0.63 | 0.13 | 0.13 | 74.3 ± 0.1 |
| 0.13 | 0.13 | 0.63 | 0.13 | 72.8 ± 1.3 |
| 0.13 | 0.13 | 0.13 | 0.63 | 69.0 ± 1.2 |
| 0.25 | 0.25 | 0.25 | 0.25 | 89.8 ± 0.3 |

**Table S15: :** Experimental results for Glc optimization obtained from digestion of AHP-treated corn stover (0.125g H_2_O_2_/g biomass, 15% solids loading, 48 hr, controlled pH) with mixtures of four commercial enzyme preparations (see Table 2). All enzyme loadings were 15 mg protein/g glucan.

| Enzyme proportions | | | | Glc yield, % |
| --- | --- | --- | --- | --- |
| Accellerase 1000 | Multifect- Xylanase | Multifect-Pectinase | Novozyme 188 |
| 1.00 | 0.00 | 0.00 | 0.00 | 62.5 ± 1.3 |
| 0.00 | 1.00 | 0.00 | 0.00 | 8.0 ± 0.2 |
| 0.00 | 0.00 | 1.00 | 0.00 | 16.0 ± 0.5 |
| 0.00 | 0.00 | 0.00 | 1.00 | 9.0 ± 0.0 |
| 0.50 | 0.50 | 0.00 | 0.00 | 65.5 ± 1.5 |
| 0.50 | 0.00 | 0.50 | 0.00 | 66.2 ± 0.7 |
| 0.50 | 0.00 | 0.00 | 0.50 | 65.0 ± 1.5 |
| 0.00 | 0.50 | 0.50 | 0.00 | 17.8 ± 0.4 |
| 0.00 | 0.50 | 0.00 | 0.50 | 11.5 ± 0.2 |
| 0.00 | 0.00 | 0.50 | 0.50 | 13.7 ± 0.8 |
| 0.63 | 0.13 | 0.13 | 0.13 | 70.5 ± 1.7 |
| 0.13 | 0.63 | 0.13 | 0.13 | 58.7 ± 3.0 |
| 0.13 | 0.13 | 0.63 | 0.13 | 52.1 ± 1.9 |
| 0.13 | 0.13 | 0.13 | 0.63 | 51.9 ± 0.8 |
| 0.25 | 0.25 | 0.25 | 0.25 | 63.1 ± 2.3 |

**Table S16:** Statistical analysis for optimization of Glc from corn stover under different AHP conditions (see Table 2).

| Pre-treatment conditions | Model optimized for | p-value | F-value | R^2 | Adjusted R^2 | Predicted R^2 | Difference between Adj and Pred R^2 | Adequate Precision |
| --- | --- | --- | --- | --- | --- | --- | --- | --- |
| 0.125g H_2_O_2_/g biomass, 10% biomass loading, 24 hr, controlled pH | Glc | <0.0001 | 21.56 | 0.91 | 0.87 | 0.81 | 0.06 | 12.1 |
| 0.125g H_2_O_2_/g biomass, 10% biomass loading, 48 hr, no pH control | Glc | <0.0001 | 16.3 | 0.89 | 0.83 | 0.75 | 0.08 | 10.7 |
| 0.5g H_2_O_2_/g biomass, 15% biomass loading | Glc | <0.0001 | 17.3 | 0.89 | 0.84 | 0.76 | 0.08 | 11.1 |
| 0.5g H_2_O_2_/g biomass, 15% biomass loading | Glc | <0.0001 | 27.9 | 0.93 | 0.90 | 0.83 | 0.07 | 15.9 |
| 0.125g H_2_O_2_/g biomass, 15% biomass loading, pH controlled, 48 hr | Glc | <0.0001 | 15.3 | 0.89 | 0.83 | 0.80 | 0.03 | 10.2 |
